# Blood–Brain Barrier in Brain Tumors: Biology and Clinical Relevance

**DOI:** 10.3390/ijms222312654

**Published:** 2021-11-23

**Authors:** Francesca Mo, Alessia Pellerino, Riccardo Soffietti, Roberta Rudà

**Affiliations:** 1 Department of Neuro-Oncology, City of Health and Science, University of Turin, 10126 Turin, Italy; alessia.pellerino@unito.it (A.P.); riccardo.soffietti@unito.it (R.S.); rudarob@hotmail.com (R.R.); 2 Division of Neurology, Castelfranco Veneto and Treviso Hospitals, 31033 Treviso, Italy

**Keywords:** blood-brain barrier (BBB), neurovascular unit (NVU), brain–tumor barrier (BTB), chemotherapy, nanoparticles (NP), convection-enhanced delivery (CED), focused ultrasounds (FUS)

## Abstract

The presence of barriers, such as the blood–brain barrier (BBB) and brain–tumor barrier (BTB), limits the penetration of antineoplastic drugs into the brain, resulting in poor response to treatments. Many techniques have been developed to overcome the presence of these barriers, including direct injections of substances by intranasal or intrathecal routes, chemical modification of drugs or constituents of BBB, inhibition of efflux pumps, physical disruption of BBB by radiofrequency electromagnetic radiation (EMP), laser-induced thermal therapy (LITT), focused ultrasounds (FUS) combined with microbubbles and convection enhanced delivery (CED). However, most of these strategies have been tested only in preclinical models or in phase 1–2 trials, and none of them have been approved for treatment of brain tumors yet. Concerning the treatment of brain metastases, many molecules have been developed in the last years with a better penetration across BBB (new generation tyrosine kinase inhibitors like osimertinib for non-small-cell lung carcinoma and neratinib/tucatinib for breast cancer), resulting in better progression-free survival and overall survival compared to older molecules. Promising studies concerning neural stem cells, CAR-T (chimeric antigen receptors) strategies and immunotherapy with checkpoint inhibitors are ongoing.

## 1. Blood-Brain Barrier: Structure and Function

The brain is extremely sensitive to a wide range of circulating toxic substances, and neuronal function needs an optimal microenvironment, maintained by three main different barrier systems: the blood-brain barrier (BBB), the blood cerebro-spinal fluid barrier (BCSFB) and the meningeal barrier [1,2].

The presence of a barrier between normal brain tissue and blood was firstly hypothesized by Paul Ehrlich in the late 19th century by observing that trypan blue dye injected into the rat circulation resulted in staining the peripheral organs but not the brain and spinal cord [3]. In the following years, Goldman injected trypan blue in the CSF (cerebrospinal fluid) and demonstrated that the staining remained confined to the CNS (central nervous system) [4]. By the 1960s, the advent of electron microscopy confirmed the presence of the blood–brain barrier, which is located at the level of the brain microvessels and represents the largest interface between brain and blood, with an estimated total area between 12 and 18 m^2^ [5]. The blood–brain barrier protects the brain from pathogens and toxic substances, maintains ionic homeostasis and nutrient supply [6]. It is constituted by endothelial cells that line cerebral capillaries, while surrounding astrocytes, pericytes and microglia interact with endothelial cells and support the function of the BBB.

Endothelial cells, localized in the inner blood vessel layer, are connected by tight junctions (TJs) formed by proteins like claudin -3, -5, occludins and zonula occludens proteins -1, -2, -3. They limit the paracellular diffusion of substances, such as solutes, ions and water, and create high electrical resistance (>1800 Ω × cm^2^) to the diffusion of polar molecules [7]. There are two types of TJs: bicellular TJs at the contact of two cells and tricellular TJs at the contact of three cells. Claudin-5 and angulin-1 are important components of bicellular TJs and tricellular TJs, respectively. Other junctions, such as adherens junctions (AJ) and gap junctions (GJ), play a significant role in cellular adhesion and communication, respectively.

Furthermore, endothelial cells express polarized transport systems, leading to selective transport of molecules across the barrier.

Based on the molecular weight, small molecules are usually carried through the BBB by simple diffusion (paracellular or transcellular) along their concentration gradient. Due to the presence of TJs, very few molecules can diffuse across the paracellular space, like small lipophilic molecules (<500 Da) [8]. Some hydrophilic or lipophilic molecules or gas (oxygen, carbon dioxide) can use the transcellular route through the endothelial cells [9].

Conversely, high-weight molecules with polar characteristics require carrier-mediated transport in the form of facilitated diffusion (along concentration gradient) or active transport using ATP (adenosine triphosphate) against concentration gradient. Some examples of facilitated diffusion are the transport of hexoses, amino acids, monocarboxylic acids or fatty acids by glucose transporter isoform 1 GLUT1/SLC2A1 or large neutral amino acid transporter 1 LAT1/SLC7A5 or monocarboxylic acid transporter/MCT1 [10].

Active transport mechanisms are divided into primary active, such as efflux transport by ABC (ATP-binding cassette) transporters, and secondary active coupling the movement of an endogenous ion along concentration gradient with the movement of another molecule against its concentration gradient.

Macromolecules are transported via adsorptive-mediated transcytosis (AMT) (albumin) or receptor-mediated transcytosis (RMT) (insulin and transferrin) [6].

AMT allows the transcytosis through the BBB of large molecules not requiring interaction with a receptor. This nonspecific process is mediated by the negative charge of the surface layer of glycans and glycoproteins called the glycocalyx, which enables the binding of cationic molecules that are ultimately internalized.

Another receptor-independent endocytic route is macropinocytosis, consisting in an internalization of extracellular fluid and molecules in pinocytic vesicles with size ranging from 200 to 600 nm [11,12].

Receptor-mediated transcytosis is clathrin- or caveolae-dependent. Transferrin receptor (TfR) represents a form of clathrin-dependent transcytosis while low-density lipoprotein receptor (LDLR) is an example of caveolae-dependent transcytosis. After the interaction between ligand and receptor, clathrin-coated endocytic vesicles of 70 to 150 nm in diameter are formed [13]. Caveolae are 50–100 nm flask-shape vesicles constituted by caveolin-1. After internalization, the vesicles are trafficked to endosomes or lysosomes for metabolism or degradation [12].

Following internalization, substances can be removed from the brain parenchyma by three different ways:

1. They are returned to the blood flow through the action of active transporters: P-glycoprotein (P-gp, ABCB1), breast-cancer-resistance protein (BCRP, ABCG2) and multidrug resistance-associated proteins (MRP1, -4, -5, ABCC1, -4, -5). Efflux pumps are expressed at the luminal side of the endothelium and transport a variety of molecules with wide structural diversity, showing a significant overlap in their substrates [14].
2. They enter the cerebrospinal fluid or the lymphatic system, and then back to the blood flow.
3. They are metabolized by enzymes of phase I (monoamine oxydases A and B and Cytochromes P450) or phase II (UDP-glucuronosyltransferases and methyl transferases), which make them sufficiently polar to be excreted from the CNS by the above-mentioned pathways.

A lack of vesicular transport with decreased pinocytosis and transcytosis further restricts drug penetration into the CNS.

Ultimately, more than 98% of small molecules cannot enter the CNS. Only drugs that fit Lipinski’s rule of five (molecular weight less than 500 Da, lipophilicity, no more than five hydrogen bond donors, no more than 10 hydrogen bond acceptors, an octanol-water partition coefficient log P not greater than 5) have the prerequisites for BBB penetration [15].

Endothelial cells are surrounded and supported in their function by pericytes and astrocytes, forming the so-called neurovascular unit (NVU).

Pericytes form gap-junctions with adjacent endothelial cells, and astrocytic endfeet cover > 99% of the endothelial-pericyte cell surface [16,17]. Pericytes are contractile cells, which provide physical support and determine the vasodynamic properties of brain capillaries, thus regulating the blood flow [18]. Pericytes are essential for the creation of barrier characteristics, like the formation of TJs, and produce extracellular matrix proteins and regulate endothelial cell proliferation, migration and differentiation [19]. Furthermore, pericytes are able to modulate the BBB integrity, transcytosis rate and expression of efflux pumps.

Astrocytes are the most abundant cell type in the brain and are characterized by the expression of glial fibrillary acidic protein (GFAP). They are in contact with neuronal synaptic spaces, monitor synaptic activity and increase nutrient delivery when the metabolic demand increases. As components of the neurovascular unit, astrocytes also contribute to the regulation of the vascular tone and local blood flow [20].

The last cell types contributing to the BBB are microglia, which represent the long-living resident immune cells and account for 12–16% of the total cell population in the brain. They remove cellular debris with a process of phagocytosis and respond to inflammatory signals, especially after breakdown of BBB: activated microglia produce proinflammatory cytokines, including IL-1β and TNF-α, that enhance further BBB disruption [21,22]. The exposition to lipopolysaccharide has been shown to disrupt TJ proteins (claudin-5 and ZO-1) in vitro [23].

The basement membrane surrounding endothelial cells and pericytes is an organized protein sheet with a thickness of 50–100 nm, playing an essential role in BBB integrity [24].

The complex organization between endothelial cells, extracellular matrix, basal lamina, pericytes, astrocytes and neurons is called neurovascular unit (NVU), in which astrocytic endfeet interact with both BBB and neurons coupling neuronal components with vascular components. In fact neuronal supply for oxygen and nutrients is traduced in chemical messages by the release of neurotransmitters like glutamate and GABA (gamma-aminobutyric acid): astrocytes are able to detect glutamate and GABAergic neuronal levels and convert those signals into vasomotor commands, in order to increase blood and nutrient supply for neurons [25].

## 2. BBB Alterations in Brain Tumors

Physiologically, BBB is absent in few vascular spaces of the normal brain, like the circumventricular organs (CVOs) and choroid plexus (CP). Here, fenestrated microvessels allow diffusion of molecules to the brain parenchyma [1].

BBB may be disrupted in many pathological conditions, like brain tumors.

In both primary and metastatic brain tumors, the integrity of the BBB is altered, being more permeable and forming the so-called brain–tumor barrier (BTB). The BTB is characterized by reduction in the expression of tight junctions, induced by vascular endothelial growth factor (VEGF) secretion from tumor cells, heterogeneous pericyte coverage with increased desmin and reduced PDGFR-β expression on the cell surface, increased number of reactive astrocytes with shrinkage of astrocyte endfeet and breakdown of basal membrane [26,27]. BBB disruption in glioma is heterogeneous, occurring mainly in the core of the tumor and depends on the stage of disease. BBB disruption is a consequence of increased VEGF expression and angiogenesis in hypoxic zones, with more immature and permeable vessels within the tumor, and correlates with higher-grade of malignancy [28]. BBB disruption in glioma results in accumulation of gadolinium on MRI (magnetic resonance imaging) within tumor regions.

BBB disruption is also heterogeneous in the four histological/molecular subtypes of medulloblastoma, where endothelial cells have different grade of fenestration, thus impacting drug transcytosis and therapeutic efficacy. In the WNT subtype, the activation of the WNT/beta-catenin pathway leads to the down-regulation of some genes normally expressed in CNS endothelium (for example claudin5 and the solute carrier family 2 member 1, also named *SLC2A1*), resulting in the presence of many fenestrations in blood vessels. A downregulation of the Notch3 pathway is also observed: in fact the *Notch3* pathway interacts with the WNT pathway in the development of the CNS vasculature, and this can partially explain the branched vasculature observed in WNT-medulloblastomas [29]. On the contrary, the SHH (Sonic-Hedgehog) subtype, characterized by the overactivation of the Sonic Hedgehog pathway, has an intact BBB [29]: in fact, Sonic Hedgehog activation is probably involved in the development of the CNS vasculature and in the maintenance of its integrity [30]. The reduced permeability of BBB observed in SHH-medulloblastomas can explain the lower sensitivity and response to chemotherapy with vincristine in this group in comparison with the WNT-subtype. Among brain metastases from breast cancer, triple-negative or basal-type often have a disrupted BBB, while Human Epidermal Growth Factor Receptor 2 (HER2) positive tends to preserve BBB integrity [31]. In addition, the expression of ABC proteins on the cell surface varies in different histological subtypes of brain metastases: HER2 positive breast cancer generally express more GLUT1 and BCRP than the other subtypes.

**Figure 1:**
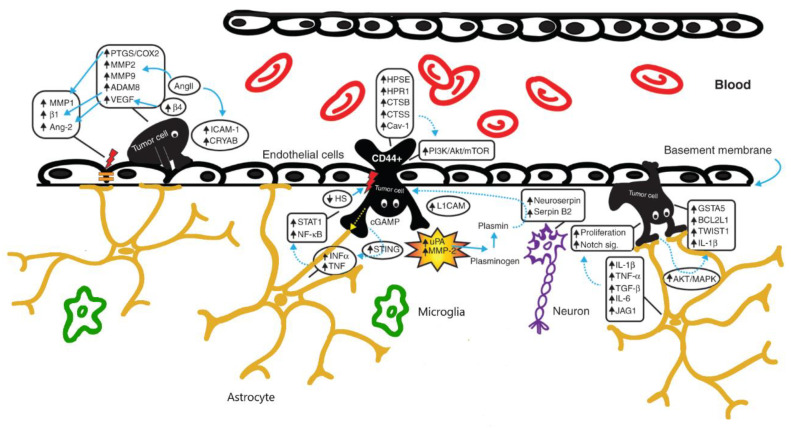
Overview of the neurovascular unit in brains harbouring tumors. The increased expression of molecules like metalloproteinases (MMP2-MMP9) and VEGF induces a breakdown of the BBB (especially the tight junctions of the endothelium) and enhances the penetration of the tumoral cells. Astrocytes contribute to the extravasation of the tumoral cells by expressing cytokines and growth factors. Modified from Pedrosa et al. [32].

In brains affected by tumors, NVU is altered in all its cellular components: in fact, besides the breakdown of the tight junctions of the endothelium, astrocytes contribute to the extravasation of the tumoral cells by expressing cytokines (especially INFα and TNF) and growth factors. Tumoral cells themselves contribute to the modification of the surrounding host tissue microenvironment (see Figure 1).

Because of this leaky BBB/BTB, many authors suggested that the BBB is no longer limiting drug delivery and hence efficacy of therapies in treating glioblastoma (GBM). However, there is increasing evidence that a heterogeneous disruption of BBB in GBM does not allow to reach homogeneous and effective drug concentrations within tumor tissue: some portions of a malignant tumor may be protected by an undamaged BBB, such as the nonenhancing regions on MRI.

For all these reasons, new drug delivery strategies to the brain should be designed to bypass an intact NVU/BBB [33].

## 3. Strategies to By-Pass BBB

A variety of strategies have been suggested to bypass the BBB and improve drug delivery in treating brain tumors. The main approaches include intratumoral, intranasal or intrathecal administration of drugs, chemical modification of molecules, chemical modulation of BBB, inhibition of efflux transporters and TJs, enhancement of transcytosis by targeting BBB transporters/receptors, physical and osmotic disruption of BBB.

### 3.1. Intratumoral, Intranasal or Intrathecal Administration of Drugs

Direct surgical injection of the drug into the brain using a cannula or intranasal delivery has been proposed.

The targets of direct surgical injections of chemotherapeutic agents are the tumor resection cavity or the surrounding brain parenchyma: this can be done via either repeated needle-based injections or catheter implants that are connected to a reservoir (i.e., Ommaya reservoir). Some studies have investigated the effect of direct intratumoral injections of carmustine and cyclophosphamide in mice, without an impact on overall survival [34]. In GBM of mice gold-iron oxide nanoparticles surface loaded with therapeutic miRNAs have been investigated: these nanoparticles were administered by intranasal route, in order to allow presensitization of GBM cells to temozolomide. A significant increase in survival was observed in the group of mice treated with nanoparticles plus temozolomide rather than in mice treated with temozolomide alone [35].

The intranasal route of nose-to-brain drug delivery can present advantages over the IV route. However, this delivery system is mainly in a preclinical phase of development.

Intrathecal administration consists in a direct injection of therapeutics into the CSF: chemotherapy may be administered directly into the lumbar thecal sac via lumbar puncture or infused into the lateral ventricles through a ventricular catheter or via the Ommaya reservoir [36].

There are several clinical reports of intrathecal administration of large molecules, including trastuzumab in patients with neoplastic meningitis from breast cancer [37,38]. However, in these studies concomitant systemic trastuzumab was administered, leading to a difficult interpretation of results. In other two studies trastuzumab was combined with cytotoxic agents, like methotrexate and/or cytarabine, with a prolongation of disease control in Her2+ brain tumor patients [39,40].

In the past, methotrexate and cytarabine (also in the liposomal form) were used by intrathecal administration in patients with neoplastic meningitis but RCTs showed no clear benefit of intrathecal administration over systemic chemotherapy (with the only exception of liposomal cytarabine in patients with lymphomatous meningitis). This was probably due to intrinsic methodological limitations such as different types of treated tumors, different chemotherapeutic agents, lack of standardization with respect to response criteria [41]. Furthermore, numerous cases of neurotoxicity and other complications like transverse myelopathy were reported [42,43]. An exception is represented by the recent randomized open-label phase III study DEPOSEIN, in which patients with neoplastic meningitis from breast cancer were randomly assigned to systemic chemotherapy versus systemic chemotherapy plus intrathecal liposomal cytarabine: an improvement of PFS (progression free survival) in the arm treated with intrathecal injections was observed [44]. However, liposomal cytarabine is no more available: thus it will not be possible to investigate a potential advantage on overall survival. In Table 1, a summary of the main routes of administration of drugs described in the text can be found.

### 3.2. Chemical Modification of Drugs

A modification of physicochemical properties of a drug to enable a passive diffusion through BBB is feasible, although few successful cases only have been reported. Chemical modification of paclitaxel, adding a succinate group at the C10 position to form the analogue Tx67, resulted in a reduction of the interaction of this compound with MDR1 (multidrug resistance type 1) in vitro and in animal models [45]. Another example of chemical modification of drugs is chlorambucil-tertiary butyl ester, a chlorambucil derivative, which achieves a sevenfold greater concentration in brain than chlorambucil [46]. More recently, etirinotecan pegol (NKTR-102) and liposomal irinotecan were tested in preclinical and clinical models (ATTAIN trial), showing increased accumulation in brain metastases and improved survival when compared to conventional irinotecan [47,48].

However, the increased lipophilicity, that enhances drug delivery to the brain, could not necessarily correlate with an increased efficacy in vivo of these compounds, due to the fact that this technique increases nonspecific binding to the brain tissue while reducing the availability for the therapeutic target.

Due to challenges in developing small CNS drugs that better cross the BBB without losing their efficacy, other approaches have been proposed, such as the encapsulation of drugs in nanoparticles (NP). Nanoparticles (NPs) loaded with antitumor drugs have been investigated to enhance the delivery of chemotherapeutic agents in patients with GBM since the early 1990s.

NPs act by prolonging blood circulation of anticancer drugs and protecting them from degradation by the reticuloendothelial system (RES). The official definition of NPs given by the European Commission is that of “an organic or inorganic object with a dimension in the range 1–100 nm”. In the biological field, NPs are considered colloidal carriers with size between 1 and 1000 nm. The drug or molecule to deliver is dissolved, dispersed, encapsulated, entrapped or attached to the NP. Many types of NPs exist with different shape, size, charge, composition and functionality. Several studies reported that NPs’ passage through the BBB is inversely related to the size [49]. An ideal NP must be nontoxic, biodegradable, biocompatible, less than 100 nm in diameter and positively charged, in order to penetrate a leaky BTB. Last but not least, it should not cause an inflammatory response. Synthetic NPs may be prepared from polymeric materials, like poly-butyl cyanoacrylate (PBCA), oligosaccharides (cyclodextrins), poly-Ɛ-caprolactone (PLC), poly-lactic-co-glycolic acid (PLGA), polyesters (poly lactic acid–PLA) or from inorganic materials like gold or silica [50]. Liposomes, micelles and extracellular vesicles are also colloidal carriers. The use of biodegradable polymers like PCL, PLA and PLGA, results in controlled drug-release lasting several days or weeks. Surface modification of nanoparticles can minimize unwanted interactions with normal tissue. The covering with polyethylene glycol can decrease the clearance of NPs by the liver, spleen and macrophages, resulting in a prolonged plasmatic half-life [51].

Substances attached on the surface of nanoparticles can target receptors or antigens on GBM cells, such as metalloproteinase-2, IL-13 receptor, integrin5β3, CD33 and CD133: the binding to these molecules leads to drug internalization by transcytosis, like a Trojan horse system.

NPs use both types of transcytosis to cross the BBB: adsorptive-mediated transcytosis (AMT) using a lectin-dependent mechanism, and receptor-mediated transcytosis (RMT) using other surface receptors like transferrin receptor (TfR) and low-density lipoprotein receptor-related proteins (LRP) by a clathrin or caveolae-dependent mechanism [52].

Liposomes represent the type of Nps, that are more commonly used, and they have a structure which is similar to that of cell membrane with high lipophilicity. Liposomes are easy to prepare and incapsulate, have high biocompatibility, and absence of significant immunogenicity.

Liposomal encapsulation of doxorubicin or taxol has proven efficacious in animal models of GBM with prolonged median survival in comparison to mice treated with the free drug [53]. In fact, doxorubicin-loaded liposomes selectively accumulate in regions of GBM with disrupted BBB. Treatment with encapsulated doxorucibin was explored in clinical trials on GBM, with a progression-free survival rate at 6 months ranging from 15% to 27% and a median overall survival (mOS) of 32–40 weeks [54,55,56]. A strategy to enhance the retention of drugs is that of targeting molecules of the exposed brain extracellular matrix (ECM) such as P1C10 (a lymphocyte receptor expressed in ECM), IL-4R and IL-13R. Neurokinin-1 receptor as well is a possible target [28].

Another approach is the use of polymeric micelles, composed of a hydrophobic polymer core and hydrophilic shell with long half-life (more than 10 h), to allow a controlled release of chemotherapeutic agents [57]. Dendrimers are the smallest molecules in use, with size less than 12 nm, and are useful for transporting short interfering RNA (siRNA) and protecting from degradation in the circulation [58]. Dendrimers loaded with methotrexate increase drug potency and efficiency in crossing BBB [59].

Metal particles were also investigated: they have a high X-Ray absorption and act as radiosensitizers. Significant DNA damage of tumor cells has been observed in animal models treated with metal particles prior to radiation therapy [60]. In Table 2, a summary of the main chemical modifications of drugs described in the text can be found.

### 3.3. Chemical Modification of BBB

Several methods of selective biochemical modulation of the BBB have been developed.

Minoxidil sulfate, a selective activator of ATP-sensitive potassium channels (K_ATP_), selectively increased BBB permeability of an experimental glioma via transcellular pathway [61]; also, an increased expression of caveolin-1 and decreased expression of TJ proteins (like occludin and claudin-5) at tumor sites was demonstrated both in vivo and ex vivo in animal models. The increased selective BBB permeability led to an improved glioma uptake of drugs of varying size, including anti-HER2 monoclonal antibody and carboplatin, and longer survival of rats.

Similarly, NS1619, an agonist of calcium-activated potassium channels (K_Ca_), injected intravenously, selectively enhanced tumor BBB permeability in glioma by modulating endocytic, transcellular processes and, when co-administered with temozolomide and trastuzumab, led to increased survival in animal models [62].

Vascular permeability is also regulated by cyclic guanosine monophosphate (cGMP): the accumulation of intracellular cGMP by inhibiting degradation with phosphodiesterase 5 inhibitor (PDE5) can result in increased permeability of brain capillaries. In rats bearing gliosarcomas both the uptake of adriamycin after oral administration of a PDE5 inhibitor (vardenafil) and survival were increased [63].

Other compounds, like bradykinin, adenosine and papaverine, were demonstrated to enhance BBB permeability and were also tested in tumor treatment. Cereport (RMP-7) is a selective bradykinin B2 receptor agonist and has been used in clinical models to trigger vasodilatation of the capillaries around brain tumors, with limited results [64].

### 3.4. Targeting of Efflux Transporters and Tight Junctions

Targeting either active efflux transporters (AET) via biochemical modifications of existing drugs or pharmacological inhibition of tight junctions (chemical or hyper-osmotic-mediated) are other novel strategies that have been explored.

Efflux transporters inhibitors have been tested both in mouse and human models in vitro. Inhibitors of Pgp, including thiosemicarbazone and tetrahydroisoquinoline derivatives, can block Pgp-mediated drug efflux in human BBB and glioblastoma stem cells [65]. Statins can reduce the activity of Pgp and BCRP by increasing the synthesis of nitric oxide (NO) [66]: this approach has yielded higher concentration of concurrently administered chemotherapeutic agents (1,5 fold for temozolomide, 5-fold for the PARP-inhibitor ABT-888 and 40-fold for vemurafenib) [67].

Claudin-5 and angulin-1 are considered candidate targets for drug delivery through the BBB [68]. Clostridium perfringens enterotoxin (CPE) can bind with high affinity to claudin-3 and claudin-4, modulating the function of the epithelial TJ barrier [69]. Another claudin-5 modulator is polyinosinic-polycytidylic acid (poly IC), a ligand of toll-like receptor 3 (TLR3), which reduces the expression of claudin-5 in a dose and time-dependent manner [70]. Bevacizumab, an antiangiogenetic agent, downregulates claudin-5 by upregulation of TGFβ1 (transforming growth factor β1) [68]. Fragments of bacterial toxins (clostridium perfringens iota-toxin) and antibodies against Angulin-1 can increase the permeability of BBB as well [71].

Intravenously injected angubindin 1, which binds to tricellular TJ protein, can increase the paracellular transport of compounds [72]. The use of interference RNA (RNAi) may reduce the expression of TJ proteins and modulate BBB permeability [73]. In the WNT subtype of medulloblastoma, the WNT-beta catenin signalling induces fenestrations in Ecs, suggesting the possible manipulation of this pathway [74].

The TJs of the cerebrovascular endothelium can be transiently and reversibly disrupted by the infusion of a hyperosmolar solution like mannitol, causing a shrinkage of the endothelial cells and an increase of paracellular diffusion of therapeutics [75]. In the clinical setting, chemotherapeutic agents used in combination with osmotic BBB disrupters (BBBD) are methotrexate, carboplatin, melphalan, cyclophosphamide and etoposide. It has been reported that osmotic BBBD plux MTX improved survival in patients with primary CNS lymphoma [76]. The infusion of a hyperosmolar solution like mannitol is nonselective and can lead to brain edema and the development of focal neurologic deficits, so this treatment modality must be weighted with the possible related side effects.

### 3.5. Physical Disruption of BBB

Another strategy to bypass the BBB consists in the physical disruption using various forms of electromagnetic radiation or ultrasounds.

Radiofrequency electromagnetic radiation (EMP) increased the permeability of BBB for compounds like Evans blue and albumin, that normally don’t cross the barrier, both in vitro and in vivo [77]. This increased permeability is transitory, appearing after 1 h, peaking at 3 h and recovering after 12 h.

Laser-induced thermal therapy (LITT) uses a stereotactically implanted laser source to induce a local, selective opening of the BBB, thus facilitating the passage of antitumor drugs, such as paclitaxel, into rat brains [78].

Conventional radiotherapy, using a dose from 20 to 40 Gy in fractions of 2 Gy, induced in glioblastoma patients a BBB opening, thus allowing a better penetration in the CNS of drugs such as methotrexate, and an increase of survival [79]. Microbeam radiation therapy (MRT), a different type of radiotherapy, rendered the tumor BBB selectively more permeable in a rat model of intracranial GBM: in fact Gd-DTPA uptake increased in the initially nonenhanced tumor area but not in the surrounding normal brain parenchyma [80]. It has been hypothesized that MRT may reduce the expression of TJ proteins, like claudin-5, ZO-1 and beta-catenin, or lead to their rearrangement [81].

Focused ultrasound (FUS) is a promising noninvasive method, associated to systemically administered microbubbles consisting of a gas core coated/encapsulated by a stabilizing shell, to enable the transient and reversible disruption of the BBB in targeted brain regions, which is restored to baseline within 6 to 24 h [82]. FUS-induced barrier opening can last for several hours depending on the molecular size of the tracer of therapeutics. The physical mechanism behind FUS-induced BBB modulation is the cavitation effect from the circulating microbubbles interacting with FUS sonication, leading to biological changes of the BBB, such as increased expression of caveolin-1 with a peak at 1 h after sonication and downregulation of TJ proteins like claudin-1, claudin-5. These alterations result in increased endocytosis/transcytosis and paracellular passage of substances.

FUS does not seem to cause ischemia in the treated regions or adverse behavioral effects; however, excessive immune reaction and brain hemorrhage caused by mechanical shear forces induced by microbubbles were described [83]. FUS parameters must be properly controlled to avoid a massive erythrocyte extravasation. Furthermore, acute inflammatory responses after treatment are described, with increase in proinflammatory cytokine genes expression at 6 h following sonication [84]. Side effects caused by FUS can be detected by MRI: FUS-induced edema is hyperintense on T2-weighted images, while T2-star sequences are able to detect red blood cell extravasation [85].

To date, FUS-mediated BBB modulation has been used for brain tumors in different CNS locations (cerebrum, brainstem and spine) to improve the penetration through BBB of antitumor drugs like doxorubicin, temozolomide, carboplatin and paclitaxel [86,87,88]. In preclinical models, FUS has been demonstrated to increase by 1.7-fold and 3.3-fold the MRI contrast enhancement in the center and margins of rat gliomas, with a prolonged median survival respect to animals treated with drugs alone [89].

Concerning clinical trials, the first feasibility and safety phase 1 study was performed between 2015 and 2017 in Canada on five patients with HGG: the procedure was well tolerated and biochemical analysis of sonicated versus unsonicated tissue confirmed the role of FUS in enhancing chemotherapy delivery [90]. Other multicentric studies showed similar results [91].

Three FDA-approved microbubbles (MBs) have been used successfully to modulate the BBB, including Definity (lipid-encapsulated microbubbles), SonoVue (sulphur hexafluoride microbubbles) and Optison (albumin coated microbubbles): Optison produced a larger effect than Definity in some studies [92], while in another one Definity and SonoVue showed similar effects [26,93].

Another strategy proposed by Bobo et al. [94] to bypass BBB in patients with glioblastoma is local drug delivery by convection-enhanced delivery (CED), which consists in the maintenance of a continuous pressure gradient and the creation of a fluid convection by the implantation into the tumor of a reservoir-catheter system, leading to the distribution of the drugs. The positive pressure created by the pump enhances the active movement of solutes and differs from passive diffusion.

CED depends on the number and position of catheters, infusion protocol, duration of infusion and type of drug infused (size, charge, lipo- or hydrophilicity). Available studies in the literature are mainly preclinical using rat models, and different therapeutic agents were infused, sometimes coated with nanoparticles or liposomes. To evaluate drug distribution, both histological and radiological exams (CT, MRI) were used. Due to the absence of strict protocols in CED use, preclinical studies investigating this technique were inconclusive.

Nowadays, no drugs have been approved for administration by CED, even though this technique was described a long time ago: unfortunately a phase III trial failed to demonstrate an efficacy of CED in drug delivery to the brain [95].

Side effects of the technique were white matter edema along the catheter tract and tissue damage with gliosis and necrosis.

In conclusion, these techniques that lead to a physical disruption of the BBB are quite invasive and may cause neurological deficits: the risks must be weighed against the potential benefits of the procedure. In this context, the use of sonicated microbubbles to modulate the BBB can produce a safe, transient and reproducible opening of the BBB, thus reducing potential side effects, being preferable over the use of CED and osmotic agents like mannitol [96].

### 3.6. The Role of Stem Cells

Lastly, a wide range of stem cell-based systems have been tested against brain malignancies, using the ability of neural stem cells (NSCs) and mesenchymal stem cells (MSCs) to cross the BBB/BTB by rolling on and adhere to endothelium with subsequent transmigration. The reason for the high tumor tropism of NSCs and MSCs are not yet fully elucidated: probably chemokines and cytokines released by the tumor may play a central role [97,98,99].

Current approaches consist in genetic modification of the carrier cell to secrete anticancer proteins, antiangiogenetic factors or immunosupportive factors like IL-12. In a recent phase I trial in glioblastoma, NSCs were modified to express an enzyme that converts a separately administered nontoxic prodrug into a cytotoxic drug [100].

In Table 3, a summary of the main mechanisms for bypassing the BBB can be found.

## 4. BBB and Drug Delivery: The Model of Brain Metastases

The issue of bypassing the BBB/BTB becomes increasingly important in brain metastases, which have a 10 times higher incidence than primary malignant brain tumors. The most frequent cancers causing brain metastases are lung cancer, breast cancer, melanoma and renal cancer, accounting for up to 80% of brain metastases [101].

In the last decade, a number of targeted therapies used in specific molecular subgroups of solid tumors were developed. The first problem to overcome is the molecular divergence, which is the difference in molecular profile between the brain metastatic and primary tumor cells that may occur in up to 50% of patients [102]. Another relevant problem is that micrometastases (<1 mm) do not alter the BBB, with a reduced efficacy of the anticancer agents employed in the adjuvant treatment [103]. This restricted entry of therapeutic agents into normal brain is one of the major contributors to the increasing incidence of brain metastases, because BBB creates a pharmacological sanctuary that protects the tumor cells to thrive in the brain [104]. For these reasons, a major challenge in the treatment of brain metastases from these tumors is the development of molecules with the ability to cross the BBB.

Concerning brain metastases from NSCLC with EGFR (epidermal growth factor receptor) mutations (mainly exon 19 deletions or L858R missense substitutions), occurring in about 10–20% of Caucasians and at least 50% of Asians [105], the efficacy of first-generation inhibitors (gefitinib, erlotinib and icotinib) is limited, because of the limited penetration into CNS of these molecules (1.3% ± 7% for gefitinib and 4.4% ± 3.2% for erlotinib) and the emergence of a second EGFR mutation on exon 20 (T790M) as a resistance mechanism. Thus, second-generation (afatinib, neratinib and dacomitinib) and third-generation (osimertinib) EGFR inhibitors have been developed. In particular, osimertinib is effective against the T790M resistance mutation and has higher BBB penetration than the first- and second-generation agents [106]. CNS objective response rate (ORR) was 91% for osimertinib versus 68% for gefitinib or erlotinib in the FLAURA trial [107]. Furthermore, osimertinib seems to exert some preventive effect with regard to the development of new brain metastases.

ALK (anaplastic lymphoma kinase) translocations are reported in 4–7% of patients with NSCLC and ALK-inhibitors showed higher efficacy in disease control compared to platinum-based chemotherapeutic schemes. The first generation ALK-inhibitor was crizotinib, but the second-generation ALK-inhibitors (ceritinib, alectinib and brigatinib) have better BBB penetration than crizotinib, with higher response rates in brain metastases (CNS response rate of 81% in the alectinib group vs. 50% in the crizotinib group, with a complete response in 38% and 5% of patients, respectively) [108]. Lorlatinib is a third generation ALK-inhibitor with high BBB penetration, which demonstrated an intracranial response of 63% after progression during treatment of at least one prior ALK inhibitor [109].

Among breast cancers, high propensity in developing brain metastases is observed among HER2 positive and triple-negative tumors. In HER2-positive tumors, the introduction in the treatment of trastuzumab (Mab against HER2) has improved the control of systemic disease and survival; however, it increased relapses in the brain consisting frequently in isolated CNS progressions, due to the low BBB penetration of the monoclonal antibody [110].

Small HER2-targeted TKIs (tyrosine kinase inhibitors) have a modest penetration across an intact BBB. For example lapatinib, an active drug against the systemic disease, showed modest penetration across the BBB and modest CNS activity, which was increased when combined with capecitabine.

Neratinib, which is an irreversible inhibitor of the HER2 family receptors, but with similar capacity to cross the BBB as lapatinib, displayed a similar activity alone or in combination with capecitabine: in the phase II trial of neratinib plus capecitabine (TBCRC 022) the objective response rate (ORR) was 49% in the lapatinib-naive cohort and 33% in the lapatinib pretreated cohort with a median PFS of 5.5 and 3.1 months respectively [111].

Tucanib is the most recent and promising HER2-targeted TKI, that led to a prolonged PFS when combined with capecitabine and trastuzumab (median PFS 7.8 months) in comparison to trastuzumab-capecitabine alone (median PFS 5.6 months) (HER2CLIMB trial) [112].

Monoclonal antibodies showed an activity on brain metastases from breast cancer, also when conjugated with other drugs (for example trastuzumab emtansine), with a demonstrated intracranial activity in preclinical and clinical models [113].

Apart from targeted therapies, new compounds derived from cytotoxic drugs with the property of better crossing of the BBB were developed, such as etirinotecan pegol (NKTR-102) and liposomal irinotecan. Both compounds achieved increased accumulation in brain metastases and improved survival in preclinical models, and in clinical trials (ATTAIN trial) when compared to conventional irinotecan [47,48]. A similar compound is ANG1005, which consists of three molecules of paclitaxel liked to Angiopep2 that enables to cross the BBB via the LPR1 (low-density lipoprotein receptor-related protein 1) transport system [114], and tesetaxel, which penetrates the intact BBB without being eliminated by P-gp [115].

In brain metastases from BRAF V600E melanoma, dabrafenib and vemurafenib, which are BRAF-inhibitors, have been extensively used, with intracranial response rates of 39.2% and 20%, respectively, with dabrafenib having a better brain distribution than vemurafenib [116]. Higher intracranial disease control was observed in patients treated with the combination of dabrafenib with trametinib (inhibitor of the MEK resistance pathway), with 78% of intracranial responses [117]. Immune checkpoint inhibitors (the anti-CTLA4 monoclonal antibody ipilimumab and the PD1 inhibitor nivolumab) showed in several studies an unrestricted access to CNS bypassing the BBB with high disease control rate, especially when used in combination [118].

Concerning renal cancer, several tyrosine kinase inhibitors (sorafenib, sunitinib and axitinib) are approved by FDA for the treatment of metastatic disease. Sunitinib showed a high brain distribution (42%) in a preclinical study, even though it is a substrate of both P-gp and BCRP [119], while brain penetration of sorafenib seems modest (9.4%) in mice, due to the efflux by P-gp and BCRP. Novel tyrosine kinase inhibitors, including cabozantinib and lenvatinib, have been approved by FDA for mRCC (metastatic renal cell carcinoma), but their capacity to cross the BBB is unknown.

## 5. Clinical Relevance and Limitations of the Current Strategies for Bypassing the BBB

Nowadays, the invasive approaches for bypassing the BBB (direct injection of molecules via intrathecal or intratumoral routes, CED) have not reached an extensive use in clinical setting.

Many chemotherapeutic agents are currently used in a lyposomal PEGylated form (doxorubicin and paclitaxel) for systemic tumors, but also for CNS tumors.

The use of focused ultrasounds (FUS), via an implantable device like Sonocloud or by using sonicated microbubbles, has shown promising results in two series of patients with glioblastoma, treated with carboplatin [120] and temozolomide/liposomal doxorubicin [90] respectively. However, this technique requires further and more detailed studies in the future and still has some important limitations. For the successful translation of this modality into humans, some unsolved technical issues must be taken into consideration, like the heterogeneity of the cranium that can cause ultrasound beam distortion and pressure attenuation, and the need for standardized ultrasound procedures [96].

## 6. Future Perspectives and Conclusions

In the last few decades, a better understanding of BBB/BTB physiology has led to the development of a multitude of strategies to target brain tumor cells; however, most of them have been tested only in pre-clinical models or in small phase 1–2 trials, and none has been approved for treatment of both primary brain tumors or brain metastases.

In the future, trials exploring the combination of new targeted therapies and strategies of BBB modulation may be useful [33].

Furthermore, better understanding of stem cell migration and stem cells’ interaction with cancer cells may lead to the development of novel strategies and therapeutic options.

Recent investigations have revealed that the disruption of the BBB in brain tumors can reduce the immunoprotective function and enhance the presentation of tumor-associated antigens and increase immune cell infiltration: as a consequence, a role of combination of immunotherapy with checkpoint inhibitors, like nivolumab, and physical approaches like FUS, has been hypothesized [121,122].

Concerning FUS, trials with Definity microbubbles at 150 µL/kg are ongoing in Canada, as well as trials with Sono-Cloud implantable devices. NaviFUS, a neuronavigation-guided FUS system, will be used in a phase I study to increase bevacizumab delivery in recurrent glioblastoma patients.

In addition, strategies that enhance the ability of T cell to penetrate the BBB/BTB, may lead to the development of CAR T (chimeric antigen receptor) strategies [123].

## Figures and Tables

**Table 1:** A summary of the main routes of administration of drugs and their clinical relevance.

Route of Administration	Type of Drug	Clinical Relevance
Intratumoral	Carmustine Cyclophosphamide	Studies in mice—no impact on OS [34]
Intranasal	Gold-iron oxide nanoparticles (plus systemic temozolomide)	Studies in mice—increased OS in the group treated with nanoparticles in addition to temozolomide respect to temozolomide alone [35]
Intrathecal	Trastuzumab ± cytarabine or methotrexateLyposomal cytarabine	No clear benefit of trastuzumab alone—better disease control when combined with cytarabine or methotrexate in Her2 breast cancers [39,40,41] Improvement in PFS when combined with systemic chemotherapy respect to chemotherapy alone [44]

**Table 2:** Some examples of chemical modification of drugs and their clinical relevance.

Mechanism of Action/Targeted Pathway	Type of Drug	Clinical Relevance
Reduced interaction with MDR1	Tx67 (paclitaxel with a succinate group in C10 position)	Animal models and in vitro—increased penetration across BBB [45]
Increased liphophilicity and plasmatic half-life	Chlorambucil-tertiary butyl ester	Animal models—higher concentrations in the brain than chlorambucil [46]
Increased plasmatic half-life and CNS penetration	Etirinotecan pegol (NKTR-102)	Studies in mice—increased overall survival compared to conventional irinotecan [47]
Increased liphophilicity	Liposomal irinotecan	Phase I study in metastatic breast cancer—intracranial objective response rate (ORR) in 30% of patients [48]
Increased plasmatic half-life and selective accumulation in GBM	Liposomal doxorubicine	Retrospective and prospective nonrandomized studies—moderate effect on PFS and OS with long-term stabilization of gliomas [54,55,56]
Increased BBB permeability and drug endocytosis	Methotrexate loaded polyether-copolyester (PEPE) dendrimers	In vitro studies—higher antitumoral activity [59]

**Table 3:** A summary of the mechanisms for bypassing the BBB.

Strategy to Bypass BBB	Mechanism Involved	Molecules Used
Direct injection of drugs	Intranasal, intratumoral (by a catheter connected to a reservoir) or intrathecal administration	Intratumoral: carmustine, cyclophosphamide [34] Intrathecal: Trastuzumab +/− cytarabine or methotrexate [39,40,41], lyposomal cytarabine [44]
Chemical modification of drugs	Conjugation with succinate or ester groups and encapsulation in nanoparticles	Tx67 (paclitaxel with a succinate group in C10 position) [45], chlorambucil-tertiary butyl ester [46], etirinotecan pegol (NKTR-102) [47], liposomal irinotecan [48], liposomal doxorubicine [54,55,56], methotrexate loaded polyether-copolyester (PEPE) dendrimers [59]
Chemical modification of BBB	Increasing BBB permeability by expression of caveolin-1 and downregulation of TJ proteins, stimulation of endocytic process, activation of cGMP and bradykinin B2 receptors	Minoxidil sulfate [61], NS1619 [62], vardenafil [63], cereport [64]
Targeting tight junctions and efflux transporters	Inhibition of Pgp and BCRP, inhibition of claudins -3, -4, -5	Thiosemicarbazone and tetrahydroisoquinoline derivatives [65], statins [66], clostridium perfringens enterotoxin (CPE) [67], polyinosinic-polycytidylic acid (poly IC) [68], bevacizumab [69], angubindin 1 [72], mannitol [75]
Physical disruption of BBB	Radiofrequency electromagnetic radiation (EMP), laser-induced thermal therapy (LITT), microbeam radiation therapy (MRT), focused ultrasound (FUS) with sonicated microbubbles (Definity, SonoVue, Optison) or implantable devices, convection-enhanced delivery (CED)	Combined treatment with chemotherapeutic drugs (paclitaxel [78], doxorubicin [86], temozolomide [87], carboplatin [88]
Stem cells	Ability to cross the BBB endothelium	Engineered to carry anticancer proteins, antiangiogenetic factors or immunosupportive factors like IL-12 [100]
